# A data‐driven investigation of relationships between bipolar psychotic symptoms and schizophrenia genome‐wide significant genetic loci

**DOI:** 10.1002/ajmg.b.32635

**Published:** 2018-04-19

**Authors:** Ganna Leonenko, Arianna Di Florio, Judith Allardyce, Liz Forty, Sarah Knott, Lisa Jones, Katherine Gordon‐Smith, Michael J. Owen, Ian Jones, James Walters, Nick Craddock, Michael C. O'Donovan, Valentina Escott‐Price

**Affiliations:** ^1^ MRC Centre for Neuropsychiatric Genetics and Genomics Cardiff University Institute of Psychological Medicine and Clinical Neurosciences Cardiff United Kingdom; ^2^ Department of Psychological Medicine University of Worcester Worcester United Kingdom

**Keywords:** bipolar, OPCRIT, psychosis, schizophrenia, sparse canonical correlation analysis

## Abstract

The etiologies of bipolar disorder (BD) and schizophrenia include a large number of common risk alleles, many of which are shared across the disorders. BD is clinically heterogeneous and it has been postulated that the pattern of symptoms is in part determined by the particular risk alleles carried, and in particular, that risk alleles also confer liability to schizophrenia influence psychotic symptoms in those with BD. To investigate links between psychotic symptoms in BD and schizophrenia risk alleles we employed a data‐driven approach in a genotyped and deeply phenotyped sample of subjects with BD. We used sparse canonical correlation analysis (sCCA) (Witten, Tibshirani, & Hastie, 2009) to analyze 30 psychotic symptoms, assessed with the OPerational CRITeria checklist, and 82 independent genome‐wide significant single nucleotide polymorphisms (SNPs) identified by the Schizophrenia Working group of the Psychiatric Genomics Consortium for which we had data in our BD sample (3,903 subjects). As a secondary analysis, we applied sCCA to larger groups of SNPs, and also to groups of symptoms defined according to a published factor analyses of schizophrenia. sCCA analysis based on individual psychotic symptoms revealed a significant association (*p* = .033), with the largest weights attributed to a variant on chromosome 3 (rs11411529), chr3:180594593, build 37) and delusions of influence, bizarre behavior and grandiose delusions. sCCA analysis using the same set of SNPs supported association with the same SNP and the group of symptoms defined “factor 3” (*p* = .012). A significant association was also observed to the “factor 3” phenotype group when we included a greater number of SNPs that were less stringently associated with schizophrenia; although other SNPs contributed to the significant multivariate association result, the greatest weight remained assigned to rs11411529. Our results suggest that the canonical correlation is a useful tool to explore phenotype–genotype relationships. To the best of our knowledge, this is the first study to apply this approach to complex, polygenic psychiatric traits. The sparse canonical correlation approach offers the potential to include a larger number of fine‐grained systematic descriptors, and to include genetic markers associated with other disorders that are genetically correlated with BD.

## 1 INTRODUCTION

Bipolar disorder (BD) is a severe, often recurrent, mental illness, associated with disability, suicide, and a reduction in life expectancy of over 10 years (Vos et al., 2015). Pervasive high mood and increased energy are core features of the disorder, characteristically alternating with spells of depression and normal mood states (Vázquez, Holtzman, Lolich, Ketter, & Baldessarini, 2015). BD is clinically heterogeneous; psychotic symptoms are present in some individuals but not others, and when these occur, they can be indistinguishable from those present in people with schizophrenia (Craddock, O'donovan, & Owen, 2005; Grande, Berk, Birmaher, & Vieta, 2016).

Molecular and epidemiological studies have reported strong evidence of shared genetic etiology between BD and schizophrenia (Andreassen et al., 2013; Cardno and Owen, 2014; Cardno, Rijsdijk, Sham, Murray, & McGuffin, 2002; Lichtenstein et al., 2009; Purcell et al., 2009; Sullivan, Daly, & O'Donovan, 2012). It is now established that common genetic variants contribute liability to both disorders, and in addition, the fraction of heritability conferred by such variants to schizophrenia and BD is substantially (around 68%) correlated (Lee et al., 2013).

A number of studies have aimed to identify characteristics of the BD phenotype that are most strongly liked to schizophrenia risk, and have generally done so by testing predefined subgroups of BD patients against total burden of schizophrenia risk alleles. Such studies have shown that in people with BD, the burden of alleles identified in studies of schizophrenia is highest in those with psychotic symptoms (Allardyce et al., 2017; Goes et al., 2012), while conversely, in people with schizophrenia, the burden of alleles identified in studies of BD is highest in people with manic symptoms (Ruderfer et al., 2014). While studies of total risk burden are providing insights into the relationships between schizophrenia and BD, a limitation of this approach is that alleles identified from studies of one disorder are considered to act uniformly on a particular symptom, or set of symptoms, in the context of people with the other disorder. Given both schizophrenia and BD are highly heterogeneous disorders, if genetic heterogeneity underpins phenotypic heterogeneity, such universal genotype–phenotype relationships are unlikely to apply.

An alternative approach is to use data‐driven approaches to seek novel relationships between phenotypic variables and genotypes. However, such analyses are challenging in the context of the high‐dimensional data, which is comprised of large numbers of associated alleles, even larger numbers of combinations of alleles, and potentially thousands of phenotypic data points and phenotypic combinations (Ehrenreich & Nave, 2014). Here, we have begun to address this problem using canonical correlation analysis (CCA) (Hotelling, 1936), an approach designed to identify linear relationships (usually hidden) between two sets of multidimensional variables. We exploit more recently developed sparse CCA (sCCA) (Witten et al., 2009), which addresses the high computation burden of CCA for high‐dimensional data by minimizing the number of features used in both phenotypic variables and genotypes while maximizing the correlation between the two sets.

The broad hypothesis underpinning our study is that schizophrenia liability is not randomly distributed in individuals with BD; rather liability is enriched among people with BD who manifest particular clinical features. We have previously shown that *en masse*, schizophrenia liability is linked to psychotic symptoms in BD (Allardyce et al., 2017). Here, we aim to extend that finding to investigate, in a purely data‐driven manner, the possibility that further granularity exists between schizophrenia liability and BD, specifically, do particular schizophrenia risk alleles (or groups of alleles) show evidence for relatively selective effects on particular psychotic features in people with BD. By way of comparison, we also undertook a phenotypic hypothesis‐based CCA, based on grouping symptoms according to a three factor classification of schizophrenia symptoms (Cardno et al., 1996).

## 2 MATERIALS AND METHODS

### 2.1 Bipolar data description

#### 2.1.1 Recruitment

Participants were available as part of the Bipolar Disorder Research Network (bdrn.org) using (a) systematic screening of community mental health teams across the United Kingdom and (b) website, media and third sector organizations. Subjects were aged 18 years or over and provided written informed consent.

Subjects were excluded if they: (a) had a lifetime diagnosis of intravenous drug dependency; (b) were judged to have only experienced affective illness as a result of alcohol or substance dependence; and (c) had only experienced affective illness secondary to medical illness or medication. This study received Multi‐Region and Local Research Ethics Committee (MREC and LREC) approvals.

#### 2.1.2 Diagnostic assessments

Information was collected by interviewing participants with the Schedules for Clinical Assessment in Neuropsychiatry (Wing et al., 1990). Psychiatric and general practice case notes were also reviewed. Interview and case note data were combined. Participants were diagnosed using DSM‐IV criteria, including 2,628 cases with BD‐I, 1,089 cases with BD‐II, 124 cases with Schizoaffective BD, and 66 cases with BD NOS. Fifty‐three percent patients with psychotic features. Clinical ratings were made according to the OPCRIT (OPerational CRITeria) checklist (McGuffin, Farmer, & Harvey, 1991). Originally designed to facilitate a polydiagnostic approach to psychotic and mood disorders for molecular genetic research, OPCRIT includes items on psychopathology and history. For the current analyses we used items concerning psychotic symptoms, rated on a lifetime‐ever basis (summarized in Table 1). Team members involved in the interview, rating, and diagnostic procedures were all fully trained research psychologists or psychiatrists.

**Table 1:** Description of OPCRIT measurements

		Full sample (*N* = 4,589)		Cleaned sample (*N* = 3,903)			
OPCRIT number	Description	Missingness (%)	Presence (%)	Missingness (%)	Presence (%)	Groups defined by schizophrenia factor analysis	Clusters defined according to a phenomenological approach
29	Third person auditory hallucinations	7.3	2	3.2	1.7	Factor 1	Cluster 1
30	Running commentary voices	6.7	0.8	2.6	0.7	Factor 1	Cluster 1
31	Abusive/accusatory/persecutory voices	8.8	7	2.6	7	Factor 1	Cluster 1
32	Other (nonaffective) auditory hallucinations	16.5	6	8.6	6	Factor 2	Cluster 1
33	Nonaffective visual hallucinations	15	3.7	9	4	Factor 1	Cluster 1
34	Nonaffective hallucination in any other modality	7.8	2	4.2	1.8	Factor 1	Cluster 1
35	Thought echo	2	0.2	0.3	0.1		Cluster 1
36	Thought insertion	3.6	0.5	1.7	0.5	Factor 1	Cluster 1
37	Thought broadcast	2.9	0.2	1	0.2	Factor 1	Cluster 1
38	Thought withdrawal	2.5	0.15	0.6	0.2	Factor 1	Cluster 1
39	Delusions of passivity	3.9	0.4	2	0.4	Factor 1	Cluster 1
40	Delusions of influence	17.2	32.5	10.4	34	Factor 1	Cluster 1
41	Primary delusional perception	2	0.15	0.2	0.2		Cluster 1
42	Persecutory delusions	12	18	6.7	19	Factor 1	Cluster 1
43	Bizarre delusions	3.6	0.9	1.3	1	Factor 1	Cluster 1
44	Other primary delusions	3.3	1	1.2	1.3		Cluster 1
45	Bizarre behavior	4	11	1.4	10	Factor 3	Cluster 3
46	Catatonia	2.4	0.3	0.6	0.4	Factor 2	Cluster 2
47	Speech difficult to understand	3	3	1.3	2.7	Factor 3	Cluster 3
48	Incoherent form of thought	3	0.3	1.2	0.3		Cluster 3
49	Positive formal thought disorder	4.3	0.4	2.3	0.4	Factor 3	Cluster 3
50	Negative formal thought disorder	3	0.4	1	0.4	Factor 2	Cluster 2
51	Restricted affect	2.2	1.5	0.4	1.4		Cluster 2
52	Blunted affect	2	0.2	0.2	0.2	Factor 2	Cluster 2
53	Inappropriate affect	2.3	2	0.5	1.7	Factor 3	Cluster 3
54	Perplexity	5.6	5	4	4.8		Cluster 2
55	Grandiose delusions	17.3	29	11	31	Factor 3	Cluster 1
56	Delusions of guilt	12.1	3.4	8.2	3.5		Cluster 1
57	Delusions of poverty	9.8	0.1	6.9	0.2		Cluster 1
58	Nihilistic delusions	12.1	1.4	8.9	1.4		Cluster 1

Description of OPCRIT items. Columns present missingness and presence of the OPCRIT items in the full sample (*N* = 4,589) and cleaned sample (*N* = 3,903), respectively. The last two columns present two different ways of lumping OPCRIT items into three groups (groups defined by schizophrenia factor analysis; groups defined using phenomenological approach).

#### 2.1.3 Quality control for OPCRIT data

The information on OPCRIT measurements was available for 4,589 BD subjects of European ancestry. The OPCRIT items most frequently rated as present also have higher missing value rates (>10%). For the CCA we excluded subjects if they had three or more missing values among the OPCRIT items; retaining 3,903 subjects.

### 2.2 Bipolar genotype quality control

BD subjects were genotyped at three different stages of sample collection on different platforms (Supporting Information Table 1). Quality control (QC) was performed on each cohort separately using PLINK2 software (Chang et al., 2015). SNPs were excluded if their minor allele frequency (MAF) <0.01; call rate <0.98, or they deviated from Hardy–Weinberg equilibrium (HWE) at *p* ≤ 10^−6^. Individuals were removed if they had genotype call rates <0.98, increased or decreased heterozygosity of |F| > 0.1, genotyped/reported sex discrepancy, high pairwise relatedness (pi‐hat 0.2), or were population outliers as identified by principle components (PCs) analysis in a joint analysis of 2,000 subjects from 19 different populations taken from the 1000 Genome project (The 1000 Genomes Project Consortium, 2015).

Following QC, the data for each cohort were imputed using SHAPEIT (Delaneau & Marchini, 2014) and IMPUTE2 (Howie, Fuchsberger, Stephens, Marchini, & Abecasis, 2012) with the 1000 Genome reference panel Phase 3, 2014. Imputed data were converted to the most probable genotypes with probability ≥0.9, and then merged. SNPs were further excluded out if their imputation INFO score was <0.8, MAF < 0.01, and HWE *p*‐value < 1 × 10^−6^).

**Figure 1:**
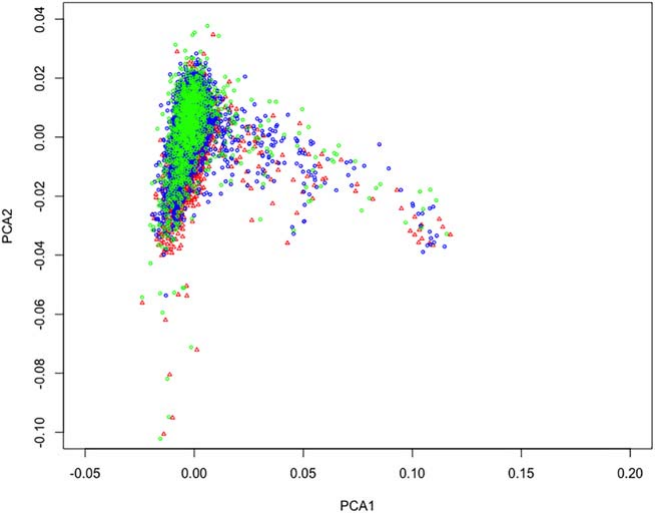
PC analysis (the first two components) for BD case genotype data. Each point represents an individual which belongs to one of the three waves of genotyping (red—WTCCC, blue—BDRN wave 1, green—BDRN wave 2) [Color figure can be viewed at http://wileyonlinelibrary.com]

To remove SNPs due to genotyping platform difference, SNP frequencies were compared in each pair of cohorts (with logistic regression) and removed if their frequencies were significantly different (*p* < .01). Our final data set contains 3211519 imputed SNPs. Ten PCs were generated and used to control for population stratification (see Figure 1 for first two PCs). The total number of individuals after QC was 3,903.

### 2.3 SNP selection

Imputed genotypes were clumped for linkage disequilibrium (LD; window 1,000 kb, *r* ^2^ = .2) using PLINK2 (Chang et al., 2015) retaining the SNPs most significantly associated with schizophrenia (The Psychiatric Genomics Consortium, 2014). We excluded the MHC region because of its complex long‐range LD properties (Price et al., 2008) and discarded schizophrenia associated SNPs for which variation in imputation across arrays resulted in low‐quality data. We retained 82 LD independent SNPs for further analysis (Supporting Information Table 2). SNPs were coded using the additive genetic model, 0 for major allele homozygous status, 1 for heterozygous, and 2 for minor allele homozygous, and adjusted for 10 PCs to control population stratification.

## 3 METHODS

### 3.1 Canonical and sparse canonical correlations

CCA captures the linear relationship between two sets of variables. CCA finds two sets of basis vectors for two sets of variables, such that the correlations between the projections of the variables onto the space spanned by the basis vectors, are mutually maximized (Hotelling, 1936). The dimensionality of these new bases is equal to, or less than, the smallest dimensionality of the two sets of variables, in our case, the minimum numbers of phenotypic variables and SNPs.

Formally, the CCA concept can be described as follows. Consider *n* subjects with two sets of multidimensional measurements, phenotypes (number of measured phenotypes is p) and genotypes (number of SNPs is equal to q). Then X is an (n×p) matrix of phenotypes for each individual and Y is an (n × q) matrix of genotypes. To quantify the relationship between them, CCA identifies vectors u and v that maximize the correlation cor(Xu, Yv). Vectors u and v are called canonical variates, which are simply linear combinations of the phenotype variables on the one side and genotypes on the other side. The canonical variates can be interpreted as factor loadings.

Extension of CCA to sCCA makes the technique more suitable for analyzing large correlated datasets (e.g., when p + q exceeds n). sCCA aims to find the “sparse” solution, that is, those projections that depend on a small number of variables, making the analysis more robust and powerful (Witten et al., 2009).

Similar to ordinary CCA, sCCA searches for canonical variates **u** and **v** that maximize the correlation cor(Xu, Yv) with additional convex penalty functions $P_{1}(u)\leq c_{1}$, $P_{2}(v)\leq c_{2}$. Parameters $c_{1}$ and $c_{2}$ give the numbers of variables for X and Y that have nonzero weight. To understand how well the sCCA captures the relationship between the two matrices, *p*‐values are usually computed using a permutation approach. In brief, computation of sCCA is performed in three stages. First, sCCA is run with a permutation option where the best parameters $c_{1}$ and $c_{2}$ are chosen based upon *p*‐values. Second, sCCA is run with the best coefficients c1 and c2 from stage 1. Third, variables for the X and  Y matrices, with nonzero weights derived in the final analyses, are extracted.

In the last decade, a number of sparse CCA approaches have been introduced. The techniques introduced by Waaijenborg, Verselewel de Witt Hamer, and Zwinderman (2008), Parkhomenko, Tritchler, and Beyene (2009), and Witten et al. (2009) impose covariance restrictions. Wilms et al. (2016) suggested that variables are selected (find a sparse solution) using a penalized regression framework. Wilms et al. (2016) demonstrated that this method outperforms CCA and some other sparse CCA approaches in almost all simulated scenarios. This methodology is freely available in package “PMA” in R on CRAN, and we chose to use it for the analyses in this study. We used 1,000 permutations to select the sparsity parameters c1 and c2. We report results only for the first sCCA dimension, since it captures the most of the variation (similar to the first PC in a standard PC analysis) and is most robust to sparsity parameters choice (Grellmann et al., 2015).

### 3.2 Imputation of phenotypes and genotypes

The proportion of missing data directly affects the quality of (s)CCA analyses as individuals with missing values are usually removed and as a consequence, power is decreased. There is no established cutoff for missing value thresholds suitable for all datasets; some suggest that 5%–10% missingness is acceptable, depending on the patterns of the missing data (Tabachnick & Fidell, 2007), others have applied a 15%–20% threshold for genomic data (Lin et al., 2013), and 20% for clinical data (Bennett, 2001).

In our data, after QC, the missing value rate does not exceed 11% for phenotypes and 5% for genotypes (see Table 1). To retain those surviving QC, we further imputed both genotype and phenotype data as implemented in the R‐package “mice.” Function mice() is a Markov Chain Monte Carlo method that uses the correlation structure of the data and imputes missing values for each incomplete variable a number of times by regression of incomplete variables on the other variables iteratively. This method benefits from automatic identification of the variable type (binary, categorical, or continues) and treats data accordingly.

### 3.3 OPCRIT groups of symptoms

**Figure 2:**
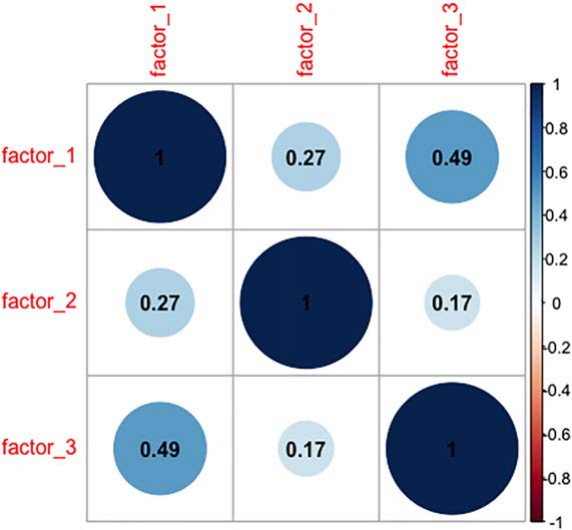
Correlation matrix between OPCRIT groups of symptoms defined by schizophrenia factor analysis (see Table 1): “factor 1,” “factor 2,” and “factor 3” [Color figure can be viewed at http://wileyonlinelibrary.com]

OPCRIT items were combined to generate three groups of symptoms: “factor 1,” “factor 2,” and “factor 3” (Table 1), as suggested by schizophrenia factor analysis (Cardno et al., 1996). In this way, the sCCA may be more powerful since the number of phenotype variables (30) is reduced, there are no missing values, the ambiguity of the imputation is minimized, and the frequencies of symptom “presence” in each the group are increased, as compared to individual OPCRIT item frequencies. Groups were coded as 0/1, where 1 represents that at least one OPCRIT symptom is present in a group. The numbers of people with the present symptom in each group were 1,655, 261, 1,385 for “factor 1,” “factor 2,” “factor 3” groups, respectively. Note that an individual can be assigned to more than one group and 1,895 subjects did not belong to either of the groups above. The correlation structure of the three dimensions is depicted in Figure 2.

To investigate our results further, we also explored a three‐cluster model based on phenomenological approach, grouping the items in three clusters, see Table 1: “cluster 1” (including positive symptoms, present in 1,964 participants), “cluster 2” (including negative symptoms, present in 247 participants), and “cluster 3” (including disorganized symptoms, present in 480 participants).

## 4 RESULTS

First, we performed sCCA for 82 genome‐wide significant (GWS) SNPs and 30 individual OPCRIT symptoms. The results are summarized in Table 2. Phenotypes and genotypes with nonzero weights chosen by sCCA are shown in columns “phenotypes” and “SNPs,” respectively. Weights can be interpreted as unstandardized regression coefficients and can be negative or positive (Supporting Information Table 2). If weights for phenotype variables and SNP variables are of the same sign that indicates that both variables are positively correlated. If the weights are of opposite sign, then they are inversely correlated. Those variables were identified from a single multivariate analysis, and therefore no multiple testing corrections to the *p*‐values are required. sCCA for individual psychotic items identified significant association between delusions of influence, bizarre behavior, grandiose delusions, and rs11411529 as in the single nucleotide polymorphism database (dbSNP) (http://www.ncbi.nlm.nih.gov/projects/SNP), also reported as indel chr3:180594593, build 37, see Supporting Information Table 2, (The Psychiatric Genomics Consortium, 2014). Delusions of influence, grandiose delusions had the largest contribution to the sCCA loadings (Supporting Information Table 3).

**Table 2:** sCCA results for 82 schizophrenia GWS SNPs

	Correlation	*p*‐value	Phenotypes chosen by sCCA	SNPs chosen by sCCA
Individual OPCRIT items	0.07	0.033	Delusions of influence, Bizarre behavior, Grandiose delusions	rs11411529
OPCRIT groups defined by schizophrenia factor analysis	0.063	0.012	“factor 3” group	rs11411529

*p*‐Values are obtained by 1000 permutations. sCCA results for GWS schizophrenia SNPs and two types of phenotypes used in the analysis (individual OPCRIT items and OPCRIT groups of symptoms). “Correlation” and “*p*‐value” columns give the best sCCA correlation coefficient and corresponding *p*‐value obtained by 1000 permutations. Columns “phenotypes chosen by sCCA” and “SNPs chosen by sCCA” show phenotypes and SNPs with nonzero weights chosen by the analysis.

The analysis of grouped OPCRIT items revealed a significant association between the “factor 3” group and the same SNP, rs11411529. As shown in Table 1, the “factor 3” group includes both grandiose delusions and bizarre behavior. A post hoc within case logistic regression analysis confirmed association between the “factor 3” group and rs11411529 (*p* = 9.1 × 10^−5^, OR = 0.79). The direction of the association was such that the schizophrenia (SZ) risk allele was associated with membership of this group, and is in agreement with the direction identified by sCCA.

As a further test, we applied sCCA to a randomly chosen half of the sample. The results were similar, rs11411529 and “factor 3” group were identified as significantly correlated (*p* = .036), a finding that replicated in the second (independent) half of the sample using logistic regression (*p* = .03; OR = 0.83).

As an exploratory analysis, we performed sCCA using sets of SNPs that are expected to be enriched for true associations to schizophrenia, but for which the evidence for association does not meet the definition of genome‐wide significance (Supporting Information Table 4). The “factor 3” group was consistently identified as the only group that correlated with schizophrenia risk alleles, and rs11411529 remained the main contributor to that association (Supporting Information Table 5). When GWS SNPs were excluded, sCCA analysis found no significant canonical correlations.

We then tested the association between genotype and three clusters of symptoms, defined using a phenomenological approach. sCCA detected a borderline significant association (*p* = .052) between “cluster 1” (which included both delusions of influence and grandiose delusions), and the same SNP rs11411529. The sCCA analysis did not identify significant association when using SNPs on less significant schizophrenia associated *p*‐value thresholds, see Supporting Information Table 4.

The sCCA for individual OPCRIT items did not identify a significant association with additional, less significant schizophrenia associated SNPs (Supporting Information Table 4). Note that in the top section of the Supporting Information Table 4, although the resulting canonical correlations are not negligible (maximum 0.33), they are not significant; this is likely due to the penalty that comes from a large number of variables (similar to the penalty for the numbers of degrees of freedom in other statistical tests).

## 5 DISCUSSION

BD and schizophrenia are distinct categorical entities according to current diagnostic systems. Nevertheless, the two disorders share many clinical features—for example, up to 50% of patients with BD present with symptoms that are common in schizophrenia such as persecutory delusions, auditory hallucinations, experiences of influence, and catatonic symptoms (Pope & Lipinski, 1978) and it is now clear their genetic etiologies also substantially overlap. The relationships, if any, between the genetic and clinical overlaps are unclear, although recent studies suggest schizophrenia risk is particularly elevated in people with BD and mood incongruent psychotic features (Allardyce et al., 2017; Goes et al., 2012).

Seeking to identify novel genotype–phenotype links, we have applied sCCA to a well‐phenotyped and genomically informative sample. sCCA is a data‐driven approach that can estimate the strength of the relationships between two sets of variables (in our example, genotypes and phenotypes); in doing so, sCCA has the potential to identify novel genotype–phenotype links without investigators imposing highly specific hypotheses. To our knowledge this is first study of its type.

The primary finding of the hypothesis‐free analysis was that a cluster of symptoms comprising the most common delusions in our sample (grandiose, of influence, as well as bizarre behavior) are particularly associated with a schizophrenia risk allele. Note that this association is a “within case” association, and this allele has not been reported as GWS associated with BD in any case‐control analysis. The association was primarily driven by a single variant rs11411529, which tags a locus spanning three genes, *CCDC39*, *DNAJC19*, and *FXR1*. It is as yet unclear which (if any of these three) confer is involved in schizophrenia susceptibility.

A second analysis in which we impose a structure to the BD phenotype based upon factor analysis of symptoms in schizophrenia identified the same allele to be associated with “factor 3” group. Being constrained, the latter analysis does not fully exploit the potential of sCCA, but the reduced dimensionality of that analysis enhances power, allowing us to detect associations once again between “factor 3” group and a larger number of SNPs based upon more relaxed significance criteria. It should be noted that sets of SNPs at those sub GWS significant thresholds are nevertheless enriched or true associations, indeed among the eight SNPs with a threshold P = 10^−5^ from the PGC (The Psychiatric Genomics Consortium, 2014) that together show significant evidence for association with disorganized features, 5 map to loci that are GWS in a larger recent schizophrenia GWAS dataset (Pardiñas, 2018); in addition to rs11411529 these include; rs999494 (EMX1); rs75968099 (TRANK1); rs6803008 (FOXP1); rs5004844 (CNTN4). The TRANK1 locus has previously been reported to be significant in a case‐control study of BD (Chen et al., 2013), and the index SNP rs75968099 is also significant in the GWAS (The Psychiatric Genomics Consortium, 2014), from which we selected alleles to be tested in this study. We speculate that the inclusion of this SNP as contributing to a multivariant association involving relaxed significance thresholds, but not the more stringent GWS threshold, possibly indicates joint association with other SNPs. We denote these above loci by gene name, but as for rs11411529, the functional basis for the associations is not understood. Further studies to confirm these associations are needed, and if confirmed, their biological functions may potentially offer a route into understanding heterogeneity of BD.

We tested the validity of sCCA to identify genotype–phenotype relationships by applying it to a random draw of half of the sample. Our finding of association between rs11411529 and “factor 3” group in the discovery half of the sample was independently replicated by a different analytic method (logistic regression) in the second (independent) half of the sample, supporting the hypothesis that sCCA can identify true associations in the complex datasets, although at present, in genomics terms, the findings are modest and need to be replicated.

Using a phenomenological approach to group OPCRIT items, we also replicated the association between the cluster of symptoms containing grandiose delusion and delusions of influence and SNP rs11411529, confirming that this association is driven mainly by these two OPCRIT items.

Strengths of this study are the use of a validated assessment tool and the assessment of inter‐rater variability (Di Florio et al., 2013); the largest sample size to date with this of granularity of phenotypic information; and phenotypic data obtained from multiple sources including case notes. Limitations are reliance on retrospective assessment of psychosis, the low prevalence of some psychotic symptoms, and missingness. In addition, the sCCA approach may not be the most powerful when genotype–phenotype relationships are nonlinear, and our sample size that while large for this type of study, is still small in the genomics context.

In summary, we show that sCCA approach is capable of revealing relationships between complex phenotype and genotype data, and provide evidence for associations between sets of SNPs and features of the bipolar phenotype. Given sample size limitations, the specific associations are best regarded as hypothesis generating, and require evaluation in other well‐phenotyped samples.

## CONFLICT OF INTEREST

None.

## Supporting information

Additional Supporting Information may be found online in the supporting information tab for this article.

Supporting Information Tables
