# Supplementary material for: A data‐driven investigation of relationships between bipolar psychotic symptoms and schizophrenia genome‐wide significant genetic loci
Source: Am J Med Genet B Neuropsychiatr Genet. 2018 Apr 19;177(4):468–75. doi: 10.1002/ajmg.b.32635 (PMC6001555; doi:10.1002/ajmg.b.32635)
Supplement: Supplementary file 1 — Supporting Information Tables [file AJMG-177-468-s001.docx]

Supplemental Table 1. Description of genotyping platforms for the BD samples.

|  | **N cases** | **Chip Name** | **N of SNPs** |
| --- | --- | --- | --- |
| **Wellcome Trust Case-Control Consortium (WTCCC)** | 1868 | GeneChip 500K Mapping Array Set (Affymetrix) | 377742 |
| **Bipolar Disorder Research Network -wave 1*** | 2577 | Omni-Express | 393635 |
| **Bipolar Disorder Research Network -wave 2** | 1104 | PsychChip | 578318 |
| * Part of the International Cohort Collection for Bipolar Disorder (ICCBD) |  |  |  |

Supplemental Table 2. GWS schizophrenia variants that have been used in the sCCA.

“CHR”, “SNP’ and “BP” are chromosome, variant rs ID, and human genome build 37 positions. “A1” and “A2”, “OR”, “p-value” show the alleles for these variant, odds ratio for allele A1 and p-value as reported in schizophrenia PGC2.

| CHR | SNP | BP | a1 SZ PGC2 | a2 SZ PGC2 | OR SZ in PGC2 | p-value SZ in PGC2 |
| --- | --- | --- | --- | --- | --- | --- |
| 1 | rs140505938 | 150031490 | T | C | 0.9132 | 9.34E-10 |
| 1 | rs10803138 | 243555219 | A | G | 0.93165 | 1.79E-08 |
| 1 | rs1498232 | 30433951 | T | C | 1.0724 | 1.28E-09 |
| 1 | rs1885246 | 73793499 | T | C | 1.06727 | 1.95E-09 |
| 1 | 1_8424984_D | 8424984 | I2 | D | 1.07144 | 2.03E-09 |
| 1 | rs1702294 | 98501984 | T | C | 0.89074 | 2.79E-17 |
| 2 | 2_146436222_I | 146436222 | I2 | D | 1.08426 | 1.07E-08 |
| 2 | rs6434928 | 198304577 | A | G | 0.92682 | 1.48E-11 |
| 2 | rs6704641 | 200164252 | A | G | 1.07918 | 3.40E-08 |
| 2 | rs11693528 | 200736507 | C | G | 0.90538 | 5.62E-14 |
| 2 | rs72974269 | 225454907 | T | C | 0.93979 | 3.27E-08 |
| 2 | rs6704768 | 233592501 | A | G | 0.92886 | 3.15E-12 |
| 2 | 2_57981434_I | 57981434 | I2 | D | 1.11394 | 2.85E-09 |
| 2 | rs2176546 | 58156772 | C | G | 1.07208 | 1.84E-10 |
| 2 | rs7596038 | 58383820 | T | C | 0.93351 | 1.15E-10 |
| 3 | rs7432375 | 136288405 | A | G | 0.93109 | 5.27E-11 |
| 3 | 3_180594593_I | 180594593 | TA | T | 0.91402 | 5.35E-11 |
| 3 | rs9841616 | 181167585 | A | T | 0.92247 | 1.65E-08 |
| 3 | rs75968099 | 36858583 | T | C | 1.0822 | 3.39E-12 |
| 3 | rs2710313 | 52969529 | A | T | 1.07412 | 1.56E-10 |
| 3 | rs1353545 | 60287845 | C | G | 1.06375 | 4.26E-08 |
| 4 | rs1106568 | 176861301 | A | G | 0.93295 | 1.15E-08 |
| 4 | rs215411 | 23423603 | A | T | 1.06695 | 1.22E-08 |
| 5 | rs10515678 | 152323236 | T | C | 0.9323 | 5.59E-09 |
| 5 | rs2973155 | 152608619 | T | C | 0.93491 | 1.02E-09 |
| 5 | rs13170232 | 152899532 | A | T | 0.90719 | 3.82E-08 |
| 5 | rs4352559 | 60586625 | T | C | 0.92886 | 5.39E-12 |
| 6 | rs9398171 | 108983527 | T | C | 0.93829 | 3.37E-08 |
| 6 | rs4712936 | 25417423 | T | G | 1.21046 | 5.38E-17 |
| 6 | rs751727 | 33764158 | A | G | 0.92432 | 2.12E-08 |
| 6 | rs3798869 | 84328660 | A | G | 0.93725 | 1.15E-09 |
| 6 | rs79979354 | 96467407 | C | G | 0.88338 | 3.15E-08 |
| 7 | 7_104691378_D | 104691378 | D | I6 | 1.06737 | 5.07E-09 |
| 7 | rs13240464 | 110898915 | T | C | 1.08394 | 6.16E-13 |
| 7 | rs7801375 | 131567263 | A | G | 0.92035 | 2.26E-08 |
| 7 | rs3735025 | 137074844 | T | C | 1.06577 | 7.75E-09 |
| 7 | 7_2025096_I | 2025096 | D | I3 | 0.92256 | 6.12E-14 |
| 7 | rs6943762 | 86403263 | T | C | 1.10087 | 3.19E-09 |
| 8 | 8_111630275_D | 111630275 | I2 | D | 0.91521 | 4.64E-10 |
| 8 | rs8180995 | 143326237 | A | G | 1.07929 | 6.98E-13 |
| 8 | rs11783093 | 27425349 | T | C | 0.91833 | 2.17E-08 |
| 8 | rs6984242 | 60700469 | A | G | 0.93763 | 1.76E-09 |
| 8 | rs7819570 | 89588626 | T | G | 1.08015 | 1.90E-08 |
| 9 | rs11139497 | 84739941 | A | T | 1.07037 | 3.09E-09 |
| 10 | rs9665626 | 104300638 | C | G | 0.90312 | 4.79E-09 |
| 10 | rs11191419 | 104612335 | A | T | 0.90665 | 9.24E-18 |
| 10 | rs10883832 | 104871279 | T | G | 1.15974 | 4.30E-16 |
| 10 | rs7893279 | 18745105 | T | G | 1.11974 | 3.56E-11 |
| 11 | rs2514218 | 113392994 | T | C | 0.93034 | 4.09E-10 |
| 11 | rs7927437 | 123395987 | T | C | 1.06748 | 1.24E-08 |
| 11 | rs11219769 | 124620147 | T | G | 0.93379 | 1.72E-08 |
| 11 | 11_130749351_D | 130749351 | D | I3 | 0.93632 | 5.91E-10 |
| 11 | rs75059851 | 133822569 | A | G | 1.09615 | 1.23E-11 |
| 11 | rs11027857 | 24403620 | A | G | 1.0646 | 3.21E-09 |
| 12 | rs2851447 | 123665113 | C | G | 0.91302 | 2.19E-14 |
| 12 | rs7312955 | 123747783 | A | C | 1.07369 | 4.68E-10 |
| 12 | rs2007044 | 2344960 | A | G | 0.91247 | 2.63E-17 |
| 12 | rs2239063 | 2511831 | A | C | 1.07144 | 5.39E-09 |
| 14 | rs12887734 | 104046834 | T | G | 1.09101 | 1.17E-13 |
| 14 | rs2332700 | 72417326 | C | G | 1.07605 | 1.69E-09 |
| 15 | rs11071614 | 61851805 | T | C | 0.94092 | 8.69E-09 |
| 15 | rs147144681 | 78900908 | T | C | 1.06812 | 2.80E-08 |
| 15 | rs8042374 | 78908032 | A | G | 1.09144 | 1.87E-12 |
| 15 | rs950169 | 84706461 | T | C | 0.92386 | 7.62E-11 |
| 16 | rs12691307 | 29939877 | A | G | 1.07315 | 1.30E-10 |
| 16 | rs12325245 | 58681393 | A | T | 0.91631 | 1.15E-08 |
| 16 | rs2018916 | 63700508 | T | C | 0.94158 | 1.26E-08 |
| 16 | rs9922678 | 9946319 | A | G | 1.06962 | 6.72E-09 |
| 17 | rs8082590 | 17958402 | A | G | 0.93557 | 6.84E-09 |
| 17 | rs7216638 | 2156453 | A | T | 0.93305 | 2.26E-09 |
| 18 | 18_52749216_D | 52749216 | I2 | D | 1.07509 | 1.75E-11 |
| 18 | rs78322266 | 53063676 | T | G | 1.19363 | 1.10E-08 |
| 18 | rs72930759 | 53153294 | T | C | 1.17328 | 2.87E-10 |
| 18 | rs9636107 | 53200117 | A | G | 0.92663 | 9.09E-13 |
| 18 | rs587136 | 53225049 | T | C | 1.08883 | 2.25E-09 |
| 18 | rs147232866 | 53621557 | A | G | 1.14042 | 1.42E-08 |
| 19 | rs2315279 | 19479936 | A | C | 0.93876 | 1.00E-08 |
| 19 | rs2053079 | 30987423 | A | G | 0.92932 | 3.79E-09 |
| 20 | rs6065094 | 37453194 | A | G | 0.92849 | 5.52E-11 |
| 22 | rs5995756 | 40000313 | T | C | 1.07498 | 2.84E-11 |
| 22 | rs35333608 | 41570145 | I2 | D | 1.06983 | 1.32E-08 |
| 22 | rs134873 | 42657566 | T | G | 0.93866 | 2.40E-09 |

Supplemental Table 3. Weights for phenotypes and SNPs that are chosen by sCCA running for 82 schizophrenia GWS SNPs and individual OPCRIT items.

| **Individual OPCRIT items** | | | |
| --- | --- | --- | --- |
| **Phenotype** | **Weight** | **SNP** | **Weight** |
| Delusions of influence | 0.65 | rs11411529 (TA/T) | -1 |
| Bizarre behaviour | 0.25 |  |  |
| Grandiose delusions | 0.74 |  |  |
|  |  |  |  |
| **OPCRIT groups defined by schizophrenia factor analysis** | | | |
| **Phenotype** | **Weight** | **SNP** | **Weight** |
| "factor 3" group | -1 | rs11411529 (TA/T) | 1 |
| **OPCRIT clusters defined by phenomenological approach** | | | |
| **Phenotype** | **Weight** | **SNP** | **Weight** |
| "cluster 1" | -1 | rs11411529 (TA/T) | 1 |

Supplemental Table 4. sCCA results for schizophrenia SNPs with less stringent thresholds.

Results are shown when analyzing individual OPCRIT phenotypes as well as grouped OPCRIT by symptoms (using schizophrenia factor analysis and phenomenological approach) and schizophrenia SNPs available in BD taken the p-value thresholds 1x10-5, 1x10-4, 1x10-3. “Correlation” and “p-value” columns gives the best sCCA correlation and corresponding p-value obtained by 1000 permutations. Columns “phenotypes chosen by sCCA” and “SNPs chosen by sCCA” show phenotypes and number of SNPs with non-zero weight chosen by the analysis.

| **Individual OPCRIT items** | | | | |
| --- | --- | --- | --- | --- |
|  | **Correlation** | **p-value** | **Phenotypes chosen by sCCA** | **SNPs chosen by sCCA** |
| **SZ p value <1e-5; 377 SNPs** | 0.23 | 0.33 |  |  |
| **SZ p value <1e-4. 902 SNPs** | 0.21 | 0.17 |  |  |
| **SZ p value <1e-3. 2558 SNPs** | 0.25 | 0.47 |  |  |
|  |  |  |  |  |
| **OPCRIT groups defined by schizophrenia factor analysis** | | | | |
|  | **Correlation** | **p-value** | **Phenotypes chosen by sCCA** | **SNPs chosen by sCCA** |
| **SZ p value <1e-5; 377 SNPs** | 0.1 | 0.038 | "factor 3" group | 8 |
| **SZ p value <1e-4; 902 SNPs** | 0.22 | 0.005 | "factor 3" group | 56 |
| **SZ p value <1e-3; 2558 SNPs** | 0.34 | 0.05 | "factor 3" group | 124 |
|  |  |  |  |  |
| **OPCRIT groups defined by phenomenological approach** | | | | |
|  | **Correlation** | **p-value** | **Phenotypes chosen by sCCA** | **SNPs chosen by sCCA** |
| **SZ p value <1e-5; 377 SNPs** | 0.088 | 0.8 |  |  |
| **SZ p value <1e-4; 902 SNPs** | 0.14 | 0.688 |  |  |
| **SZ p value <1e-3; 2558 SNPs** | 0.23 | 0.241 |  |  |

Supplemental Table 5. Weights for phenotypes and SNPs that are chosen by sCCA for schizophrenia SNPs with less stringent threshold and grouped OPCRIT items by schizophrenia factor analysis.

Columns “Phenotypes chosen by sCCA”, “SNPs chosen by sCCA” and “Weight” are phenotypes and SNPs chosen by sCCA with corresponding non-zero weights. “CHR”, “BP”, “A1” and “A2” are chromosome, variant rs ID, human genome build 37 position, as it appears in BD sample. The results presented in batches for different schizophrenia p-value thresholds of 1x10-5, 1x10-4, 1x10-3.

| **schizophrenia p-value<1e-5** | | | | | | | |
| --- | --- | --- | --- | --- | --- | --- | --- |
| **Phenotypes chosen by sCCA** | **Weight** | **CHR** | **SNPs chosen by sCCA** | **BP** | **A1** | **A2** | **Weight** |
| "factor 3" group | -1 | 3 | rs11411529 | 180594593 | TA | T | 0.796 |
|  |  | 7 | rs1734907 | 100315517 | A | G | 0.302 |
|  |  | 3 | rs5004844 | 2568588 | T | C | -0.489 |
|  |  | 3 | rs6803008 | 71571345 | T | C | -0.0573 |
|  |  | 16 | rs71391092 | 74567075 | G | C | 0.0714 |
|  |  | 2 | rs7577463 | 228994782 | T | G | 0.032 |
|  |  | 3 | rs75968099 | 36858583 | T | C | -0.0324 |
|  |  | 2 | rs999494 | 73157395 | T | C | 0.162 |
| **schizophrenia p-value<1e-4** | | | | | | | |
| "factor 3" group | -1 | 11 | 11_124992779_D | 124992779 | G | GT | 0.312 |
|  |  | 11 | 11_45319967_I | 45319967 | ATT | A | 0.00739 |
|  |  | 13 | 13_80056765_D | 80056765 | AG | A | 0.00669 |
|  |  | 3 | rs11411529 | 180594593 | TA | T | 0.425 |
|  |  | 3 | 3_71676427_I | 71676427 | T | TC | 0.0592 |
|  |  | 14 | rs10131244 | 93853569 | G | A | -0.24 |
|  |  | 5 | rs10463223 | 92320089 | G | A | -0.00479 |
|  |  | 12 | rs10783580 | 53847473 | A | G | 0.121 |
|  |  | 11 | rs10891354 | 112263503 | T | C | -0.0142 |
|  |  | 9 | rs10975956 | 6997045 | A | G | 0.0346 |
|  |  | 13 | rs11841483 | 70705442 | G | C | -0.0311 |
|  |  | 1 | rs12144576 | 115361733 | A | C | -0.117 |
|  |  | 5 | rs12186681 | 90940109 | C | T | -0.0146 |
|  |  | 12 | rs12301769 | 72231313 | C | A | 0.0055 |
|  |  | 7 | rs13240464 | 110898915 | C | T | 0.0179 |
|  |  | 1 | rs139283246 | 97120607 | G | C | 0.0489 |
|  |  | 19 | rs141958336 | 2165383 | A | G | 0.00218 |
|  |  | 8 | rs1434281 | 103671080 | A | G | -0.00159 |
|  |  | 7 | rs1734907 | 100315517 | A | G | 0.223 |
|  |  | 10 | rs181975957 | 105367026 | A | G | 0.0716 |
|  |  | 22 | rs182427987 | 41485593 | T | C | -0.0963 |
|  |  | 12 | rs1860341 | 3716852 | T | C | -0.362 |
|  |  | 12 | rs1867521 | 23585643 | G | A | -0.0267 |
|  |  | 13 | rs2314449 | 56821623 | T | C | 0.0045 |
|  |  | 21 | rs35434802 | 25028377 | A | T | -0.349 |
|  |  | 3 | rs3796186 | 36876154 | T | C | 0.0164 |
|  |  | 1 | rs3900555 | 61063225 | G | A | 0.00605 |
|  |  | 4 | rs4594699 | 47264659 | T | C | -0.1 |
|  |  | 12 | rs4759527 | 129879459 | A | G | -0.201 |
|  |  | 20 | rs4814463 | 16223808 | T | C | 0.0396 |
|  |  | 4 | rs4860200 | 59867437 | G | T | -0.0985 |
|  |  | 3 | rs5004844 | 2568588 | T | C | -0.3 |
|  |  | 4 | rs5013969 | 19150738 | T | C | 0.0288 |
|  |  | 20 | rs55851153 | 58269521 | C | G | 0.059 |
|  |  | 6 | rs563440 | 151288991 | C | T | -0.0268 |
|  |  | 22 | rs5749696 | 22063337 | T | C | 0.122 |
|  |  | 22 | rs5761662 | 27050953 | A | G | -0.0355 |
|  |  | 20 | rs6065777 | 43669641 | G | T | 0.0224 |
|  |  | 3 | rs62283301 | 177379760 | A | G | -0.00882 |
|  |  | 13 | rs66786230 | 44711625 | C | T | -0.0227 |
|  |  | 3 | rs6803008 | 71571345 | T | C | -0.122 |
|  |  | 10 | rs7079338 | 106043132 | T | C | 0.0689 |
|  |  | 11 | rs7113199 | 134247187 | A | C | -0.0152 |
|  |  | 11 | rs7116939 | 83204454 | C | T | -0.112 |
|  |  | 16 | rs71391092 | 74567075 | G | C | 0.128 |
|  |  | 6 | rs74990847 | 108174152 | G | A | 0.09 |
|  |  | 2 | rs7577463 | 228994782 | T | G | 0.112 |
|  |  | 2 | rs7590938 | 217463442 | G | A | 0.0138 |
|  |  | 3 | rs75968099 | 36858583 | T | C | -0.112 |
|  |  | 15 | rs782934 | 61362500 | G | C | 0.0627 |
|  |  | 5 | rs79430854 | 155094642 | A | G | 0.078 |
|  |  | 17 | rs8066384 | 78625756 | T | C | 0.042 |
|  |  | 7 | rs844753 | 71534558 | G | T | -0.0655 |
|  |  | 13 | rs9515090 | 110286542 | A | G | -0.0886 |
|  |  | 17 | rs9908102 | 12896553 | C | T | 0.0452 |
|  |  | 2 | rs999494 | 73157395 | T | C | 0.165 |
| **schizophrenia p-value<1e-3** | | | | | | | |
| "factor 3" group | -1 | 11 | 11_124992779_D | 124992779 | G | GT | 0.222 |
|  |  | 11 | 11_73819189_D | 73819189 | C | CTG | -0.0975 |
|  |  | 2 | 2_40481542_D | 40481542 | T | TTG | -0.0457 |
|  |  | 3 | rs11411529 | 180594593 | TA | T | 0.307 |
|  |  | 3 | 3_71676427_I | 71676427 | T | TC | 0.0314 |
|  |  | 7 | 7_132819541_I | 132819541 | A | C | -0.0511 |
|  |  | 7 | 7_154992897_D | 154992897 | T | TG | -0.0715 |
|  |  | 9 | 9_102869231_D | 102869231 | A | AAAG | 0.0144 |
|  |  | 8 | rs10108708 | 143102886 | G | A | 0.0479 |
|  |  | 14 | rs10131244 | 93853569 | G | A | -0.168 |
|  |  | 6 | rs1072904 | 4298125 | C | T | -0.00169 |
|  |  | 12 | rs10783580 | 53847473 | A | G | 0.078 |
|  |  | 11 | rs10892957 | 122907942 | T | C | 0.104 |
|  |  | 9 | rs10975956 | 6997045 | A | G | 0.0129 |
|  |  | 5 | rs111410415 | 31226474 | A | G | -0.000748 |
|  |  | 11 | rs111433147 | 57324364 | C | G | 0.123 |
|  |  | 6 | rs111606477 | 49774744 | A | G | 0.137 |
|  |  | 6 | rs114022810 | 17016276 | T | C | -0.022 |
|  |  | 7 | rs11767718 | 44553492 | T | C | 0.165 |
|  |  | 13 | rs11841483 | 70705442 | G | C | -0.0102 |
|  |  | 14 | rs11844549 | 47310983 | C | T | 0.0163 |
|  |  | 8 | rs11985002 | 104386138 | A | G | 0.0753 |
|  |  | 1 | rs12025891 | 237992408 | A | G | -0.0973 |
|  |  | 1 | rs12031155 | 53714139 | T | C | 0.0229 |
|  |  | 1 | rs12144576 | 115361733 | A | C | -0.0752 |
|  |  | 1 | rs12145625 | 5684788 | A | G | 0.0271 |
|  |  | 6 | rs12207480 | 74003384 | T | A | 0.0248 |
|  |  | 10 | rs12252305 | 105279335 | A | G | 0.0439 |
|  |  | 11 | rs12422015 | 57193653 | G | A | -0.0455 |
|  |  | 7 | rs12702149 | 45615783 | T | C | 0.0251 |
|  |  | 7 | rs13240464 | 110898915 | C | T | 0.000273 |
|  |  | 2 | rs13426837 | 60570629 | A | G | 0.0395 |
|  |  | 1 | rs139283246 | 97120607 | G | C | 0.0236 |
|  |  | 7 | rs1406684 | 86090906 | A | G | -0.201 |
|  |  | 4 | rs141277912 | 55705026 | A | G | 0.00663 |
|  |  | 7 | rs142422599 | 108653179 | C | T | -0.0715 |
|  |  | 9 | rs143696033 | 1507663 | A | G | 0.0417 |
|  |  | 2 | rs150595656 | 119888724 | C | A | 0.0121 |
|  |  | 7 | rs1528355 | 121143380 | A | T | -0.0318 |
|  |  | 6 | rs163996 | 94037427 | G | T | -0.0284 |
|  |  | 9 | rs1666625 | 114711109 | G | A | 0.088 |
|  |  | 3 | rs17323271 | 60027584 | G | A | -0.112 |
|  |  | 7 | rs1734907 | 100315517 | A | G | 0.155 |
|  |  | 8 | rs17363324 | 77624803 | T | C | 0.128 |
|  |  | 14 | rs1741235 | 95361114 | T | A | -0.113 |
|  |  | 10 | rs181975957 | 105367026 | A | G | 0.0408 |
|  |  | 22 | rs182427987 | 41485593 | T | C | -0.0594 |
|  |  | 12 | rs1860341 | 3716852 | T | C | -0.26 |
|  |  | 12 | rs1867521 | 23585643 | G | A | -0.00694 |
|  |  | 9 | rs2025278 | 89921808 | C | G | -0.0472 |
|  |  | 20 | rs2179706 | 56139871 | T | C | -0.0362 |
|  |  | 9 | rs2253928 | 130228569 | C | G | 0.115 |
|  |  | 7 | rs2402669 | 123297460 | C | G | 0.102 |
|  |  | 12 | rs2405619 | 86148347 | C | A | 0.0173 |
|  |  | 17 | rs2440132 | 67397935 | G | A | 0.000742 |
|  |  | 3 | rs2567343 | 197192462 | A | G | -0.0221 |
|  |  | 8 | rs2634448 | 18443397 | A | T | 0.0543 |
|  |  | 1 | rs2786738 | 199557772 | C | T | -0.0195 |
|  |  | 9 | rs28712486 | 86481873 | A | C | -0.0896 |
|  |  | 8 | rs3115770 | 118743647 | AA | TA | -0.0543 |
|  |  | 5 | rs34304490 | 57503265 | G | C | -0.00337 |
|  |  | 1 | rs34593358 | 150686633 | T | A | 0.0726 |
|  |  | 21 | rs35434802 | 25028377 | A | T | -0.25 |
|  |  | 2 | rs3811570 | 153484685 | A | G | -0.0208 |
|  |  | 5 | rs4512122 | 157588362 | A | C | -0.0271 |
|  |  | 4 | rs4594699 | 47264659 | T | C | -0.0625 |
|  |  | 1 | rs4652813 | 183534544 | C | G | -0.0852 |
|  |  | 3 | rs4682944 | 44229735 | T | C | -0.0928 |
|  |  | 6 | rs4713812 | 34528955 | T | C | 0.0429 |
|  |  | 12 | rs4759527 | 129879459 | A | G | -0.138 |
|  |  | 20 | rs4814463 | 16223808 | T | C | 0.0166 |
|  |  | 4 | rs4860200 | 59867437 | G | T | -0.061 |
|  |  | 3 | rs489779 | 191181215 | C | G | -0.0353 |
|  |  | 3 | rs5004844 | 2568588 | T | C | -0.213 |
|  |  | 4 | rs5013969 | 19150738 | T | C | 0.0085 |
|  |  | 20 | rs55851153 | 58269521 | C | G | 0.0313 |
|  |  | 6 | rs563440 | 151288991 | C | T | -0.00696 |
|  |  | 22 | rs5749696 | 22063337 | T | C | 0.0785 |
|  |  | 22 | rs5761621 | 26999358 | G | A | -0.0287 |
|  |  | 22 | rs5761662 | 27050953 | A | G | -0.0136 |
|  |  | 20 | rs6020911 | 49629479 | T | C | -0.0249 |
|  |  | 20 | rs6065777 | 43669641 | G | T | 0.00368 |
|  |  | 2 | rs60960031 | 21530659 | A | G | 0.0489 |
|  |  | 12 | rs61938054 | 119571771 | A | G | -0.0661 |
|  |  | 18 | rs62086080 | 9454429 | A | G | -0.047 |
|  |  | 7 | rs62474683 | 115020725 | G | A | -0.106 |
|  |  | 6 | rs6568683 | 111815015 | A | C | -0.0778 |
|  |  | 1 | rs6671986 | 215387822 | T | C | -0.0712 |
|  |  | 13 | rs66786230 | 44711625 | C | T | -0.00387 |
|  |  | 1 | rs6680131 | 74106106 | A | G | 0.0833 |
|  |  | 1 | rs6701883 | 175294963 | G | T | 0.158 |
|  |  | 2 | rs6725177 | 29007855 | C | G | -0.0379 |
|  |  | 2 | rs6727488 | 196371240 | C | T | 0.182 |
|  |  | 3 | rs6803008 | 71571345 | T | C | -0.079 |
|  |  | 8 | rs6994092 | 76419145 | T | C | 0.114 |
|  |  | 10 | rs7074107 | 2189971 | A | G | 0.00244 |
|  |  | 10 | rs7079338 | 106043132 | T | C | 0.0387 |
|  |  | 11 | rs7116939 | 83204454 | C | T | -0.0709 |
|  |  | 16 | rs71391092 | 74567075 | G | C | 0.0833 |
|  |  | 1 | rs71656158 | 97122864 | G | A | -0.0912 |
|  |  | 14 | rs72687614 | 47929214 | C | T | 0.0537 |
|  |  | 11 | rs72869844 | 24514399 | C | G | -0.173 |
|  |  | 13 | rs7335833 | 66833474 | A | T | -0.0487 |
|  |  | 11 | rs734434 | 125018740 | A | G | -0.157 |
|  |  | 10 | rs74465643 | 105219172 | T | C | 0.146 |
|  |  | 6 | rs74990847 | 108174152 | G | A | 0.0546 |
|  |  | 2 | rs7577463 | 228994782 | T | G | 0.0712 |
|  |  | 3 | rs75968099 | 36858583 | T | C | -0.0713 |
|  |  | 13 | rs78197225 | 70622177 | A | G | -0.0584 |
|  |  | 15 | rs782934 | 61362500 | G | C | 0.034 |
|  |  | 9 | rs78697939 | 72076445 | G | C | 0.0834 |
|  |  | 7 | rs79114312 | 67216834 | T | C | 0.0676 |
|  |  | 5 | rs79430854 | 155094642 | A | G | 0.0456 |
|  |  | 7 | rs79990269 | 86324855 | A | G | -0.0232 |
|  |  | 17 | rs8066384 | 78625756 | T | C | 0.0185 |
|  |  | 7 | rs844753 | 71534558 | G | T | -0.0362 |
|  |  | 21 | rs845946 | 32706350 | A | G | -0.0904 |
|  |  | 2 | rs847138 | 176901200 | G | A | 0.000725 |
|  |  | 13 | rs9515090 | 110286542 | A | G | -0.0536 |
|  |  | 4 | rs956601 | 27597775 | T | C | 0.0371 |
|  |  | 17 | rs9908102 | 12896553 | C | T | 0.0209 |
|  |  | 18 | rs9951562 | 53452446 | A | G | -0.0809 |
|  |  | 2 | rs999494 | 73157395 | T | C | 0.111 |
